# Differences in MPS I and MPS II Disease Manifestations

**DOI:** 10.3390/ijms22157888

**Published:** 2021-07-23

**Authors:** Christiane S. Hampe, Brianna D. Yund, Paul J. Orchard, Troy C. Lund, Jacob Wesley, R. Scott McIvor

**Affiliations:** 1 Immusoft Corp., Seattle, WA 98103, USA; jake.wesley@immusoft.com; 2 Department of Pediatrics, University of Minnesota, Minneapolis, MN 55455, USA; bdy@umn.edu (B.D.Y.); orcha001@umn.edu (P.J.O.); lundx072@umn.edu (T.C.L.); 3 Immusoft Corp., Minneapolis, MN 55413, USA; r.scott.mcivor@immusoft.com or; 4 Department of Genetics, Cell Biology and Development and Center for Genome Engineering, University of Minnesota, Minneapolis, MN 55413, USA

**Keywords:** mucopolysaccharidosis type I, mucopolysaccharidosis type II, glycosaminoglycans, dermatan sulfate, heparin sulfate

## Abstract

Mucopolysaccharidosis (MPS) type I and II are two closely related lysosomal storage diseases associated with disrupted glycosaminoglycan catabolism. In MPS II, the first step of degradation of heparan sulfate (HS) and dermatan sulfate (DS) is blocked by a deficiency in the lysosomal enzyme iduronate 2-sulfatase (IDS), while, in MPS I, blockage of the second step is caused by a deficiency in iduronidase (IDUA). The subsequent accumulation of HS and DS causes lysosomal hypertrophy and an increase in the number of lysosomes in cells, and impacts cellular functions, like cell adhesion, endocytosis, intracellular trafficking of different molecules, intracellular ionic balance, and inflammation. Characteristic phenotypical manifestations of both MPS I and II include skeletal disease, reflected in short stature, inguinal and umbilical hernias, hydrocephalus, hearing loss, coarse facial features, protruded abdomen with hepatosplenomegaly, and neurological involvement with varying functional concerns. However, a few manifestations are disease-specific, including corneal clouding in MPS I, epidermal manifestations in MPS II, and differences in the severity and nature of behavioral concerns. These phenotypic differences appear to be related to different ratios between DS and HS, and their sulfation levels. MPS I is characterized by higher DS/HS levels and lower sulfation levels, while HS levels dominate over DS levels in MPS II and sulfation levels are higher. The high presence of DS in the cornea and its involvement in the arrangement of collagen fibrils potentially causes corneal clouding to be prevalent in MPS I, but not in MPS II. The differences in neurological involvement may be due to the increased HS levels in MPS II, because of the involvement of HS in neuronal development. Current treatment options for patients with MPS II are often restricted to enzyme replacement therapy (ERT). While ERT has beneficial effects on respiratory and cardiopulmonary function and extends the lifespan of the patients, it does not significantly affect CNS manifestations, probably because the enzyme cannot pass the blood–brain barrier at sufficient levels. Many experimental therapies, therefore, aim at delivery of IDS to the CNS in an attempt to prevent neurocognitive decline in the patients.

## 1. Introduction

Mucopolysaccharidosis type I and II are two of eleven inherited lysosomal storage diseases associated with disrupted glycosaminoglycan catabolism.

Mucopolysaccharidosis type II (MPS II) [1] is a genetic disorder inherited in an X-linked recessive pattern and affects ~1/160,000 live male births [2]. However, the prevalence of MPS II shows geographical differences, and MPS II is the more common form in East Asia, while MPS I is the more prevalent form in Europa and North America [3]. MPS II is caused by deficiency of the lysosomal enzyme iduronate 2-sulfatase (IDS). IDS catalyzes the first step of the degradation of heparan sulfate (HS) and dermatan sulfate (DS). IDS deficiency results in the accumulation of these glycosaminoglycans (GAGs). This causes lysosomal hypertrophy and an increase in the number of lysosomes in cells. GAGs are present throughout the body and are an essential part of the extracellular matrix (ECM). Lack of GAG turnover in the ECM affects cellular functions, like cell adhesion, endocytosis, intracellular trafficking of different molecules, intracellular ionic balance, and inflammation. Despite MPS II being the most variable and wide-ranging of the MPS types, MPS II is often described as either an attenuated or “non-neuronopathic” phenotype, or a severe, progressive, “neuronopathic” phenotype involving central nervous system dysfunction. However, patients with less severe neurocognitive disease may still exhibit severe somatic symptoms, calling the term “attenuated disease” into question [4,5]. Moreover, the disease encumbers a broad spectrum of cognitive and somatic features, so that the distinction into two separate phenotypes may not be accurate [5,6].

Approximately 2/3 of patients with MPS II develop CNS manifestations [7,8,9,10], which become evident at 2–4 years of age [11]. Symptoms progress with age, and death in untreated patients typically occurs by 10–15 years of age [12]. Patients with lack of or mild neurological involvement typically display symptoms at a later age and often survive into their 5th decade [10].

The closely related disease mucopolysaccharidosis type I (MPS I) is caused by deficiency of the lysosomal enzyme iduronidase (IDUA), catalyzing step two in the degradation of HS and DS. In both MPS I and MPS II, HS and DS accumulate and patients share characteristic phenotypical manifestations, including worsening skeletal disease reflected in short stature, inguinal and umbilical hernias, hydrocephalus, hearing loss, coarse facial features, protruded abdomen with hepatosplenomegaly, and neurological involvement with varying functional concerns (Table 1). Manifestations are more prevalent and severe in the most severe form of MPS I (Hurler syndrome or MPS IH), which constitutes >50% of MPS I cases [13,14]. Overall, manifestations shared by both MPS I and MPS II usually develop earlier in children with severe MPS I as compared to MPS II (Table 2) [11,12,15,16,17,18]. A few MPS I and MPS II specific manifestations have been described [19]. In this review, we will discuss these differences and the consequences on treatment options, and we speculate about possible biochemical mechanisms for these differences.

## 2. Accumulated Glycosaminoglycans

Since the enzymatic defects in MPS I and MPS II both result in the accumulation of HS and DS, the clinical differences between both diseases cannot be explained by GAG species accumulation alone. However, biochemical analyses of HS and DS levels in urine and tissue samples of patients with MPS I and MPS II suggest distinct differences in the relative HS and DS levels, sulfation pattern, and non-reducing terminus status [42].

### 2.1. DS/HS Ratio

Overall, patients with MPS II present with higher HS levels, while patients with MPS I show higher DS levels [43]. Langereis reported that, even though the levels can vary considerably between patients, the urinary DS/HS ratio in patients with MPS I is consistently above 1, while it is consistently below 1 in patients with MPS II [44]. Other reports confirmed lower DS/HS ratios in patients with MPS II compared with patients with MPS I [45,46]. Significantly higher HS/DS ratios were also observed in MPS II mice [47,48]. Importantly, these urinary DS/HS ratios reflect those in CSF and brain [47,49].

The possible outcomes of the different ratios of HS and DS on the respective pathogenesis are demonstrated in two other MPS types, MPS III and MPS VI. Patients with MPS III show accumulation of HS only and suffer from neurodegeneration, seizures, and diarrhea, while coarse facial features, hepatomegaly, hernias, and dysostosis multiplex are milder in form [50]. Patients with MPS VI accumulate DS only and present with severe dysostosis multiplex, joint stiffness, coarse facial features, and corneal clouding, in the absence of impaired intellectual development 51]. Consistent with these studies, a correlation between elevated DS levels (relative to HS levels) can be found in patients with MPS with severe viscera and skeletal manifestations [49,52], while elevated HS levels (relative to DS levels) are associated with dysfunction of the CNS [43,52,53,54,55]. Indeed, MPS II patients with neurocognitive involvement showed significantly elevated HS levels in their CSF compared with patients without cognitive impairment [56].

### 2.2. HS and DS Functions

HS is a major component of the ECM and plays a critical role in cell signaling, recognition of growth factors, cytokines, morphogens [57,58], and regulation of chemokine and cytokine gradients [59,60]. Abnormal HS levels in the CNS lead to dysregulation of neuronal differentiation, growth, and neurotransmission [42,61,62], which may, in part, explain the higher prevalence of neurocognitive involvement in patients with MPS II.

DS is found in skin, bones, connective tissues, and cartilage, with biological functions including binding of growth factors, cytokines, and chemokines [63]. Its role in skeletal growth and development of the cornea has been illustrated in rats treated with the antiviral drug Tilorone. Tilorone treatment is accompanied by accumulation of DS, with only minor HS accumulation [64,65]. The treated rats develop bone alterations and corneal clouding similar to those seen in MPS I [66,67].

### 2.3. Sulfation Levels

**Figure 1:**
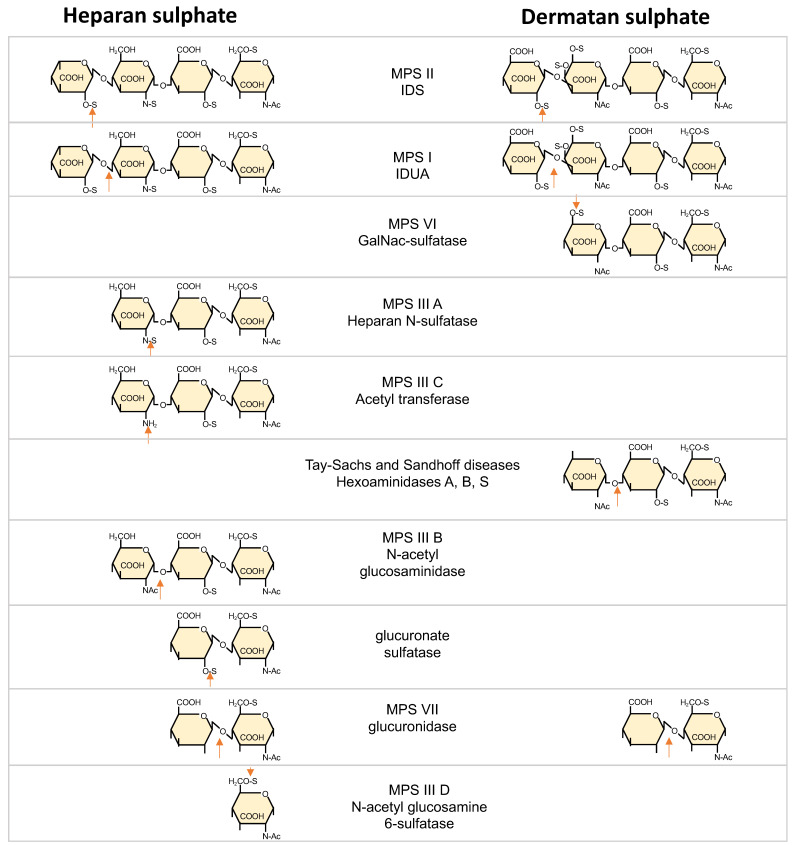
GAG catabolism begins with endohydrolysis of the polysaccharide chains to oligosaccharides. After this initial step, HS and DS oligosaccharides are degraded from their nonreducing ends (NRE) to monosaccharides and inorganic sulfate. The enzymes involved in the first two steps are IDS, which hydrolyzes the C2-sulfate ester bond of nonreducing terminal alpha-L-Iduronic acid (IdoA) residues in HS and DS and IDUA, which removes non-sulfated terminal alpha-L-IdoA residues. Under normal conditions, HS degradation produces IdoA and glucuronic acids (GlcA), while DS degradation results in IdoA and N-acetyl galactosamines (GalNAc). Defects in IDS activity result in accumulation of oligosaccharides with sulfated IdoA units on their NRE, while defects in IDUA activity result in oligosaccharides with non-sulfated IdoA units at their NRE [69].

Apart from different DS/HS ratios, MPS I and MPS II present different degradation products of both GAGs [68]. These differences stem from the position of IDS and IDUA in the GAG degradation pathway, respectively (Figure 1).

Differences in sulfation degrees and patterns were reported in 1975 by Ramage and Cunningham [70]. Later analyses of fibroblasts isolated from patients with MPS I and unaffected human donors for HS degradation products revealed the presence of non-sulfated IdoA in patients with MPS I only. These NRE species were also found specifically in the urine of patients with MPS I. Likewise, the analysis of fibroblasts isolated from patients with MPS II revealed sulfated IdoA [71]. HS disaccharides with increased levels of sulfation are found in both MPS I and MPS II mice [42,72,73].

Patients with MPS suffering from joint contractures, skeletal deformities, and cardiac valvular thickening, but without CNS involvement showed lower levels of sulfated HS, supporting a connection between CNS manifestations and HS sulfation degree and pattern [68]. This led to the hypothesis that the presence of partially degraded HS with sulfate moieties at their NRE (as expected in MPS II) may be associated with CNS manifestations, while the lack of chemical moieties at the NRE (as in MPS I) is not [74].

Mechanistically, it is well understood that degree of sulfation and sulfation patterns determine the binding of HS to growth factors and other proteins [75,76,77,78,79], e.g., sulfation of HS is necessary for the binding of many HS-binding cofactors, such as CXCL12 [80] and FGF [81,82].

## 3. Disease-Specific Gene Expression

Besides differences in HS and DS biochemistry, other factors may be involved in disease-specific manifestations. Recent in-depth transcriptome analysis in different MPS types identified disease-specific gene expression patterns for MPS I and MPS II [83,84,85]. Many of these genes were related to behavior, cell activation, and apoptosis. While a comprehensive analysis will be necessary to correlate specific manifestations to gene expression profiles, this is a promising approach to understand the different clinical outcomes between these different MPS types. It also remains to be established whether and how these differences in gene expression are related to the faulty GAG metabolism.

## 4. Disease-Specific Proteomics

A similar approach to disease-specific gene expression was taken when analyzing protein expression pattern in MPS I and MPS II. Proteomic analysis of urine samples may identify novel biomarkers for the diagnosis and prognosis, especially when the identified protein is associated with distinct MPS phenotypes. Two publications report a number of urinary proteins found in patients with MPS [86,87]. Some urinary proteins with upregulated expression were shared by patients with MPS I, II, and IV, while others were disease-specific [86]. Notably, patients with severe forms of MPS I or MPS II showed higher expression levels for specific proteins than their respective milder disease phenotypes. However, no overlap in the proteins identified in these two studies was observed. While the identification of disease-specific proteins in urine may lead to biomarkers that can be collected in a noninvasive manner, urinary proteins only present a section of the whole proteome. Proteomic analysis of whole MPS I mouse brain identified 50 proteins with a fold change ≥ 3.5 compared to control mice [88]. Proteomic analysis of the hippocampus of MPS I mice found 32 proteins with differential expression [89]. However, little or no overlap was observed when comparing the protein expression patterns of these two studies. Cardona et al. [90] choose a functional approach when investigating the interactive proteome of IDS in mouse brain. Using a combination of affinity purification and mass spectrometry, they were able to identify 187 IDS-binding proteins in brain tissue extracts from C57BL/6 mice.

A thorough analysis of these different proteomic studies, possibly together with the above discussed transcriptome analyses, may allow the identification of disease-specific pathways and open new treatment options.

## 5. Disease Manifestation

### 5.1. Systemic Manifestations: Skin, Kyphosis, Corneal Clouding, Valvular Heart Disease

A distinguishing feature in both severe and attenuated forms of MPS II are the presence of hypopigmented papules and nodules [10,15,29,91,92]. These skin manifestations are often present on the back, chest, neck, arms, and thighs and appear early in the disease [92,93,94]. The lesions are 2–10 mm in diameter, and can combine to form ridges or reticular patterns [29,92]. It is believed that these papules result from the coalescence of cytoplasmic vacuoles that subsequently release their mucinous contents into the extracellular space to form papules and nodules, as indicated by an abundance of extracellular deposition of metachromasia [93]. Histologically, the papules show irregularly organized collagen bundles separated by interfibrillar material [92]. While this skin manifestation is unique for MPS II, it is not exhibited in all patients with MPS II [92]. A possible link to HS accumulation is suggested by the observation of similar dermal nodules in scleromyxedema, where HS is hypothesized to promote binding of FGF1 to FGFR-1, and thereby promote fibroblast proliferation [95]. Another epidermal manifestation found mainly in patients with MPS II is Mongolian spots, which have been described in Asian patients with MPS II [30]. While the presence of Mongolian spots is common in Hispanic, Asian, and African newborns [96], they usually disappear during early childhood. In patients with MPS II this skin pigmentation persists well into late childhood [30].

**Figure 2:**
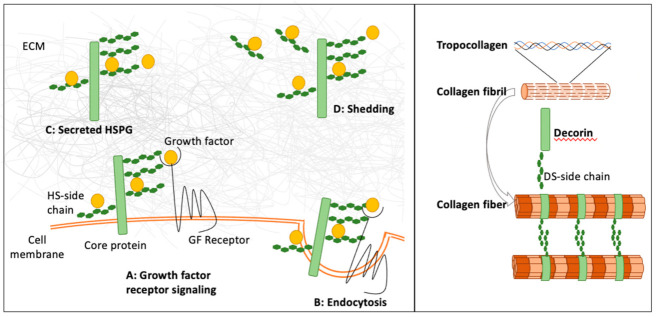
Examples of the biological functions of DS- and HS-containing proteoglycans. (**Left panel**) HSPGs affect growth factors and morphogens in a number of ways that are dependent on their sulfation pattern, their cellular localization, and the core protein [76]. HSPGs can be tethered to the cell surface (syndecan and glypicans), where they retain growth factors and activate receptor signaling (A), or mediate clearance of growth factors by endocytosis of growth factors and their receptors (B). HSPGs promote and stabilize the ternary complex between the growth factor, HS, and growth factor receptor. The formation of the ternary complex is enhanced by higher sulfation levels. Other HSPGs (perlecan and agrin) are secreted and localized in the ECM, where they can trap growth factors, and thus generate growth factor reservoirs and prevent their diffusion (C). HSPGs and HS can also be released by shedding, and thus become soluble (D). Soluble HSPGs can facilitate growth factor dispersal or movement through the extracellular space. (**Right panel**) During collagen fibrillogenesis, procollagen is synthesized as a triple helix that self-assembles into striated collagen fibrils after cleavage of the C- and N-terminal propeptides. The collagen fibrils are then arranged into uniform collagen fibers. The precise spacings between collagen fibers are maintained by the DS-containing proteoglycan decorin.

One of the most recognizable distinctions between MPS II and MPS I is the presence of corneal clouding in the vast majority of patients with MPS I, while this manifestation is mild or absent in MPS II. The stromal layer of the cornea is made up of collagens, keratocytes, and proteoglycans carrying keratin (KS) and DS side chains, while HS plays only a minor role. Keratocytes synthesize and degrade both collagens and GAGs. The highly organized arrangement of uniform collagen fibrils and fibers is critical for the transparency of the cornea and is regulated by the DS-containing proteoglycans, decorin and biglycan [97] (Figure 2). Decorin consists of a collagen-binding core protein linked to one DS side chain. Mutations in the decorin gene are associated with the development of cloudy corneas, developing shortly after birth in patients with congenital stromal dystrophy [98,99]. The respective contributions of the decorin core protein and the attached DS side chains were resolved in an elegant experiment using decorin-deficient fibroblasts transfected with GAG-free decorin. Collagen fiber diameters showed significant increases, indicating that the DS side chain of decorin controls collagen fiber diameters [100].

In MPS I, DS accumulates within the keratocytes, causing them to swell and lose their characteristic morphology [101]. Partially degraded DS also accumulates in granules throughout all corneal layers [102]. Both GAG deposits cause the disruption of the parallel arrangement of the collagen fibril, leading to corneal clouding [103,104]. The relatively lower levels of DS in MPS II may contribute to delayed development of corneal clouding, or lack thereof.

Kyphosis in MPS is characterized by poor bone growth in the anterior–superior aspect of the cranial lumbar vertebrae, resulting in anterior wedging and posterior displacement of the vertebral body [26,105]. It is unclear why kyphosis is more frequently observed in patients with MPS I. However, patients with Ehlers-Danlos syndrome (musculocontractural type 1), a disease caused by lack of dermatan sulfotransferase and subsequent abnormal DS sulfation levels [106], also characteristically develop thoracolumbar kyphosis [107]. This suggests an involvement of abnormal DS sulfation in kyphosis in patients with MPS I as well.

Cardiac valve disease is common in patients with MPS I, II, and VI [108]. The relative absence of cardiac valve involvement in MPS III (accumulation of HS only) suggests a correlation between cardiac valve dysfunction and accumulation of DS, rather than HS [109]. This perception is supported by findings of cardiac valve thickening in the majority of patients with MPS I or MPS VI [13,35,36,110,111] and only ~ 50% of patients with MPS II [10]. Mechanistically, the relative prominence of DS compared to other GAGs in cardiac valves may contribute to the underlying cause [112].

### 5.2. Neurological Involvement: White Matter Abnormalities, Neurocognitive Functioning, Behavioral Manifestations, Seizures, Sleep Abnormalities

GAG levels in the brain are significantly increased for MPS I and MPS II (up to sixfold) [113,114]. GAG deposits may impair CSF reabsorption, leading to enlarged perivascular spaces (PVS), communicating hydrocephalus and ventriculomegaly [61,115]. Indeed, central nervous system (CNS) morbidities in MPS are well known, including major structural effects, such as hydrocephalus and cervical spinal cord compression [9,116,117], as well as atrophy in some severe, progressive phenotypes [118]. Some more recent sophisticated volumetric imaging studies have also revealed other important structural findings that are less obvious in a clinical scan for a single patient, such as abnormalities in white matter volume development, particularly when compared to unaffected controls [119,120,121]. White matter involvement has been quantified in both MPS I and MPS II across the spectrum of disease. In the cognitively severe type of MPS I, white matter dysfunction is a common finding post-HSCT [122]. However, even in patients with the more attenuated type of MPS I disease without a history of HSCT, white matter abnormalities are prevalent [120]. These white matter abnormalities are also present in both the cognitively severe and attenuated forms of MPS II, indicating brain-based effects of MPS even in the “non-neuronopathic” patients.

In addition to structural abnormalities, patients with MPS also show evidence of functional CNS effects of disease, including neurocognitive deficits. In severe, “neuronopathic” MPS types, developmental delay, neurocognitive regression, behavioral changes, and sleep disturbances have been described [9,117,123]. In patients with severe MPS I, cognitive decline and/or delay in speech and language milestones is common [39]. Cognitive functioning in patients at the severe end of the MPS I disease spectrum typically reaches a plateau before 2 years of age, and is followed by rapid deterioration with aging to a profoundly impaired state by age 4 [22,124]. In contrast, in patients with severe MPS II, cognitive development reaches a plateau at around a mean age of 4–4.5 years of age, though with significant variability. Unlike the rapid intellectual loss that is evident in severe MPS I patients, the velocity of intellectual regression in the cognitively severe type of MPS II is much more complicated to calculate, due to a protracted plateau of developmental stagnation that can last years [4].

In the attenuated forms of MPS I and MPS II, somatic disease is common and is associated with poorer functioning. While many individuals with the attenuated form of MPS I have average intellect, below average cognitive functioning has been reported in a proportion, with genotype and somatic disease burden predicting neurocognitive ability [39,125,126].

Deficits in attention span, and related to white matter abnormalities, have also been reported in MPS I patients with the attenuated form of disease [120]. In patients with the less progressive, attenuated MPS II phenotype, intelligence is typically normal and remains stable; however, deficits in attention, with evidence of difficulties with executive functioning and visual-motor skills, have been noted [121]. Similar to findings in the MPS I population, there is also evidence of associations between somatic disease burden and neurocognitive functioning, namely in the areas of attention [121].

The behavioral phenotype of young patients with the cognitively severe type of MPS I has generally been described as social, compliant, and somewhat fearful [9,127,128]. These characteristics have also been corroborated in work comparing the behavioral phenotypes of young patients with MPS III to MPS I, with MPS I patients being more likely to stay close to caregivers, more likely to startle in response to loud noises, and being more compliant with caregiver commands [129]. While behavioral manifestations are not commonly seen early on, as MPS I patients age, there are emerging difficulties with attention [122,130]. Later in adolescence, there is also evidence that MPS I patients experience low self-esteem, depression, and social withdrawal. Recent work has also reported a high proportion of late-onset psychiatric manifestations, including depression, psychotic episodes (independent of depression), hyperactivity, inattention, and dementia that emerge approximately 15 years post-HSCT [131,132,133].

In contrast, patients at the cognitively severe, “neuronopathic” end of the MPS II disease spectrum demonstrate neurobehavioral symptoms that are significant and represent a characteristic feature of this phenotype [134]. Previously described as “aggressive” in early literature, accumulating research has been able to parse out these neurobehavioral symptoms to include difficulties with focus/attention, hyperactivity, impulsivity, behavior manifestations (e.g., destructiveness, aggressiveness, defiance), sensation-seeking behavior, poor emotional regulation (temper tantrums, excitable, anxious), sleep disturbance, and perseverative chewing behavior [7,8,9,12,128,135]. As such, it has been posited that the aggressive behavior often observed in children with severe MPS II may be caused by a combination of frustration, limited communication skills, anxiety, sensory-seeking behavior, and poor emotional regulation [136,137]. These symptoms generally worsen in intensity and frequency with age, until they subside with worsening overall disease progression.

Seizures are rare in patients with MPS I and, if present, are typically resolved after HSCT [38], but are frequent in MPS II patients with CNS involvement [138,139]. In contrast, seizures are not commonly reported in the patients with MPS II lacking CNS manifestations [140].

Sleep disturbances in children with MPS I appear to be related to upper respiratory manifestations, rather than behavioral issues [128], whereas, in children with MPS II, sleep disturbances are multifactorial, with one factor being CNS involvement, as they are more frequent in neuronopathic patients [128]. Many MPS II patients experienced reduced rapid eye movement sleep, night-time wakening, difficulty settling, or insomnia, which has been described as likely attributed to sleep apnea or seizure-like activity. Notably, behavioral and sleep manifestations represent early clinical markers of CNS involvement in patients with neuronopathic MPS II.

#### HS in Neurological Manifestations

HS is produced in astrocytes, neurons, and oligodendrocytes [141] and is involved in many aspects of neuronal development [142]. Based on the central role of HS in binding to growth factors and chemokines, abnormal HS sulfation may interfere with proper neuronal proliferation and survival [143,144,145]. Aberrant presentation of HS has also been implicated in the activation of neuroinflammation, which is often observed in mouse models for both MPS I and MPS II [61,72]. Mechanistically, immune responses can be activated by soluble HS acting as TLR4 agonists [146,147]. This activation appears to be further enhanced by high levels of sulfation [148].

Another potential mechanism links the neurological pathology in MPS to Alzheimer’s Disease, Huntington’s Disease, and Parkinson’s Disease [149]. HS associates with tau and alpha-synuclein and can promote their aggregation, cellular uptake, and transcellular propagation of fibrils [150,151,152]. Since sulfation of HS is necessary for tau binding [151], over-sulfated HS in MPS II may promote tau and alpha-synuclein binding, while the presence of non-sulfated HS in MPS I may be protective. This hypothesis remains to be tested.

Finally, lysosomal storage of GAG may also affect the activity of other lysosomal hydrolases, causing an increase in storage of glycosphingolipids [153,154] and gangliosides GM2 and GM3 [113,114,155]. However, cellular distributions of GAGs and gangliosides GM2 and GM3 show only limited overlap in mouse brains, so that other mechanisms may be involved in the accumulation of GM2 and GM3 [156].

## 6. Animal Models

While several animal models, including dogs, cats, and mice, exist for MPS I [157], no animal affected by MPS II was described until 1998. In 1998, a male Labrador retriever was successfully diagnosed with MPS II [158]. In this spontaneous model, the animal showed characteristic coarse facial features, with an enlarged tongue, ataxia, corneal clouding in one eye, and progressive neurologic decline. MPS II was confirmed by DNA analysis and the mother was identified as a carrier of a defective IDS gene. The exact nature of the genetic defect was not described. While several of the manifestations resembled those in children with MPS II, skeletal abnormalities were not observed. The first knock-out mouse for MPS II was generated by Muenzer et al. [159]. Since then, a number of additional knock-out mice were generated [73,160,161,162,163,164,165,166,167,168,169]. The animals are born without evident disease manifestations. While there are few differences in the age of disease onset, all MPS II mouse models show increased excretion of GAG, with GAG accumulation in the peripheral organs and the brain (Table 3). Foamy cells are observed in most tissues. The animals have coarse fur and alopecia, deformations in their joints and skeleton, with apparent thickening of the zygomatic bones, thickened digits leading to claw-like appearances of the front paws, coarse facial features, and a decline in activity.

Animal models aide in the understanding of the pathology of the disease and preclinical studies. However, species-specific disease manifestations need to be considered. Canine animal models for MPS I tend to show milder manifestations compared to humans afflicted by the disease [170,171,172]. The introduction of exact mutations in mouse models provides insights into the genetics of the disease [173,174]. However, the small size and short lifespan of mice need to be considered when evaluating therapeutic efficiency.

## 7. Natural History

Systemic and neurologic manifestations tend to appear earlier in patients with severe MPS I than in patients with severe MPS II [175], resulting in relatively late diagnosis of MPS II (Table 2). A similar trend is also observed in the mouse models for MPS I and MPS II (Table 3).

## 8. Treatment

For a comprehensive discussion of current and exploratory treatments for MPS I we refer to earlier reviews [188,189].

The FDA approved treatment of MPS II with recombinant IDS in 2006. Currently, there are two recombinant IDS enzymes available for enzyme replacement therapy (ERT): idursulfase (Elaprase^®^) or idursulfase beta (Hunterase^®^) [190]. IDS is administered weekly at 0.5 mg/kg as an intravenous infusion. ERT has significantly lowered the risk of death in patients with MPS II [31,191,192,193] and has had a beneficial effect on many of the biochemical and systemic manifestations, including reduced urinary GAG excretion levels, decreased volumes of liver and spleen, and increased cardiopulmonary function and average walking distance (Table 4) [194,195]. However, it is believed that the enzyme does not cross the blood–brain barrier (BBB) and is, therefore, not likely to have an effect on CNS disease among severely affected patients [196,197,198].

Treatment of MPS I consists of ERT in attenuated cases and hematopoietic stem cell transplantation (HSCT) in patients with severe MPS I, where early transplantation can stem many of the CNS manifestations of the disease [188]. Indeed, MPS I was the first metabolic disease to have a successful disease-modifying therapy, e.g., HSCT [226,227].

Early reports of the outcome of HSCT in patients with severe MPS II were not as promising [224,228], specifically regarding neurologic decline [178,208,209,224]. Together with these earlier reports and the risks associated with HSCT, transplants have been less common as treatment for MPS II in most Western countries. The demonstration of donor cells in the CNS of patients with MPS II that underwent HSCT caused a renewed interest in HSCT for the treatment of CNS manifestations [229]. More recent studies showed indications of improved activities of daily living and systemic manifestations (Table 4). The differences in outcomes between the original and the later studies may have been caused by suboptimal transplantation material [209,224] or more established neurological deficit at treatment in the earlier transplantations [208,209,224]. Unlike MPS I, manifestations in MPS II emerge later in life and many of the earlier transplants were administered after 2 years of age, when a beneficial effect on the CNS is less likely [178,230]. Based on the new studies and HSCT-related lowered mortality [206], HSCT is currently an accepted treatment for patients with MPS II in Japan, China, and Brazil [198,212,215,220,231]. A retrospective study of four children diagnosed with MPS II, who received HSCT, indicated overall favorable outcomes, with improved or stabilized somatic and neurocognitive manifestations [217].

### 8.1. Treatment in Animals

In a dose-finding study, the effects of systemic IDS administration at 0.1–1 mg/kg/week were investigated in MPS II mice. A dose of 0.5 mg/kg/week successfully reduced GAG levels in liver, heart, and spleen, but not in kidney or lung [232]. Treatment with 1 mg/kg/week further reduced GAG levels in kidney and lung. No significant reduction in brain GAG was achieved at any dose [232]. Tissue specific differences in IDS levels were observed with the majority of enzyme accumulating in the liver, while other tissues, such as kidneys, heart, lungs, and brain accumulated < 1% of the administered dose. [232]. Other studies reported significantly reduced HS and DS levels in all tissues, except for brain at 0.5 mg/kg/week. However, both HS and DS levels remained above those of control mice [48].

For ERT, the lack of benefit on the CNS has been largely attributed to the inability of IDS to cross the BBB. However, this point of view has been challenged by studies demonstrating that high systemic levels of enzyme can penetrate the CNS and ameliorate neurologic manifestation in MPS II animals [166,168,233]. While the IDS enzymatic activity reached only 2% of that observed in wildtype brain, GAG accumulations in brain tissues were significantly reduced [166,168,234]. Thus, the standard dose of 0.5 mg/kg/week may be too low to affect the CNS. Indeed, systemic administration of 1.2 mg/kg/week of human IDS was demonstrated to prevent progression of CNS defects in MPS II mice. Improvement was even observed in old animals (7 months) that received a high dose (10 mg/kg/week) of IDS [168]. These results were confirmed when systemic administration of 2.0 mg/kg/week recombinant human IDS resulted in significant reduction of GAG brain levels, attenuated enlarged ventricles, normalization of Purkinje cell morphology in the cerebellum, and reduction in apoptosis [235].

Delivery of IDS via adeno-associated virus (AAV) gene transfer in mice resulted in high levels of circulating IDS, with beneficial effects on systemic and CNS manifestations [162,168,234]. Another method to achieve high levels of IDS is through ex vivo genetic modification of HSC. Transplantation of lentiviral transduced HSC resulted in an increase in serum IDS levels several-fold higher than wild type [167,236]. GAG accumulation in all tested tissues, including the brain, was resolved. and deterioration of neurofunction was prevented [167]. Moreover, bone manifestations were affected with significant reduction of zygomatic arch width. In-depth analysis of bone parameters revealed higher osteoclast number, suggesting activation of bone resorption [236].

### 8.2. Experimental Therapies and Clinical Trials

The high prevalence of CNS manifestations in patients with MPS II and ineffectiveness of ERT has motivated the development of experimental therapies with the aim of IDS delivery to the brain. Early diagnosis is an essential factor to prevent CNS manifestations, as they cannot be reversed. Newborn screening has been suggested to be implemented to aide in the early diagnosis of MPS. More than 600 mutations have been identified for the IDS gene locus. Several studies investigated correlations between genotypes to phenotypes, but, to date, no clear correlation has been established [237,238]. Deletions, recombinations, frameshift, and nonsense mutations are often associated with a severe form of the disease, while most missense mutations are found in patients with attenuated disease [239,240,241,242,243].

#### 8.2.1. ERT to the Brain

Direct administration of IDS in MPS II mice via ICV or lumbar IT infusion resulted in successful delivery and morphological improvements in the brain [244]. ICV injections lowered HS levels in the CSF and in brain tissue, and yielded improved cognitive function evaluated by open-field and fear-conditioning tests [245]. This method was further advanced by enabling continuous IT infusion through an osmotic pump, which decreased GAG accumulation and vacuolization [246]. Clinical trials revealed significant reduction of GAGs in the CSF after enzyme administration via an intrathecal drug delivery device [247] (NCT00920647 and NCT00937794). An extension study (NCT01506141) to trial NCT00920647 revealed mixed results with some patients showing stable scores, while cognition worsened in others [248]. However, no beneficial effect on neurocognitive function could be established in a phase II/III study enrolling children with severe MPS II and a mild to moderate level of cognitive impairment (NCT02055118). A second phase II/III extension clinical trial (NCT02412787) [249] is currently active, but not recruiting. Recent results of a 100-week ongoing phase I/II clinical trial conducted in Japan (JMACCT CTR JMA-IIA00350) in six children with severe MPS II showed a decrease of CSF HS levels (>50% decrease) and promising effects on cognitive development [250].

#### 8.2.2. Shuttling of IDS Across the BBB

Tissue-specific delivery of peripherally administered IDS has been attempted by targeting receptors present on the luminal side of the BBB.

Systemic administration of IDS fused to an anti-human transferrin receptor antibody (JR141) in 10-week-old MPS II mice resulted in significant reduction of HS accumulation in the CNS and prevented neurodegeneration [251,252,253]. A phase I/II clinical trial (NCT03128593) in 14 patients with MPS II resulted in significant reduction of HS levels in the CSF at a dose of 2 mg/kg/week [254]. The subsequent phase II/III clinical trial (NCT03568175) not only confirmed HS level reduction, but also demonstrated improved neurocognition in 21/28 patients [255]. A Brazilian phase I/II dose-escalation clinical trial (NCT03359213) confirmed the 2 mg/kg/week dose as most effective with minimal side effects [256]. Patients showed stabilization of neurocognition and adaptive behavior, and indications for improved neurodevelopment even in older patients. An extension study for this trial (NCT03708965) is currently active, but not recruiting.

Fusing IDS to a monoclonal antibody against the human insulin receptor allowed the transport of enzyme across the BBB in rhesus monkeys [257,258]. A phase I/II clinical trial (NCT02262338) to evaluate safety of this product was completed, with no results posted yet.

#### 8.2.3. Gene Therapy: In Vivo

ICV delivery of AAV9 vector transducing the IDS coding sequence resulted in high-level IDS, both in circulation and the brain, accompanied by prevention of development of cognitive deficits observed in untreated controls [160,169,233]. Importantly AAV9 has the ability to cross the BBB [259] and has been successfully used to deliver lysosomal enzyme to the CNS in mouse models of MPS III [260,261]. Two clinical trials (NCT03566043, NCT04571970) are currently recruiting patients with MPS II for targeted delivery of the IDS gene to the CNS using AAV9.

#### 8.2.4. Genome Editing

The zinc-finger nuclease (ZFN)-mediated gene editing technology was used to insert the IDS coding sequence into the albumin locus for high-level protein expression [262]. Delivery of the construct by AAV8 vector to hepatocytes in MPS II mice resulted in significant increases in IDS activity and GAG reduction in blood and peripheral tissues and GAG reduction in the brain, which was associated with prevention of cognitive impairment [233]. A phase I/II clinical trial (NCT03041324) introducing the IDS coding sequence via ZFN-mediated genome editing resulted in reduction of GAG in the urine, although no IDS enzyme activity was detected in the plasma [263].

#### 8.2.5. Oligodendrocyte-Like Cells, DUOC-01, to Accelerate CNS Engraftment of Donor Cells

The remarkable ability of HSCT to prevent CNS manifestations in MPS is most likely due to the engraftment of donor-derived microglia or microglia-like cells in the CNS [264]. However, successful engraftment requires repopulation of the recipient myeloid compartment through donor hematopoietic progenitors [265,266], a process that takes over 6 months in transplanted mice [267]. During this time the decline in neurocognitive function continues. Thus, HSCT must be initiated well before the onset of neurologic decline. Kurtzberg et al. at Duke University developed a novel cell product, the Duke O Cell, or DUOC-01. These cells are derived from umbilical cord blood (UMCB) and efficiently treat demyelinating conditions [268]. DUOC-01 are derived from UMBC CD14+ monocytes and resemble oligodendrocyte-like cells [269]. DUOC-01 are delivered intrathecally after systemic transplantation to accelerate delivery of donor cells to the CNS, thereby bridging the gap between systemic transplant and engraftment of cells in the CNS and preventing disease progression. A phase I clinical trial (NCT02254863) is currently recruiting participants to evaluate the effect of intrathecal delivered DUOC-01 after UMCB transplantation in patients with MPS II and other inborn errors of metabolism.

## 9. Conclusions

Disease-specific manifestations of the closely related diseases MPS I and MPS II may be caused by differences in levels of accumulated storage materials and their sulfation patterns. The diseases’ major storage molecules, dermatan sulfate (DS) and heparan sulfate (HS), have unique functions, which can be further modified by sulfation levels. It remains unclear whether the apparent resistance of MPS II neurocognitive impairment to HSCT is caused by these biochemical differences, or whether early reports were artifacts of experimental design. It will be necessary to resolve this issue to determine whether patients with MPS II can be successfully treated with HSCT, or whether future therapies can be developed to ensure sufficient delivery of the therapeutic enzyme to the CNS.

## Figures and Tables

**Table 1:** Prevalence of clinical manifestations in severe types of MPS II and MPS IH in the absence of therapy.

Manifestation	MPS II (Hunter Syndrome)	References	MPS IH (Hurler Syndrome)	References
**Umbilical hernia**	70–95%	[12,19]	60–75%	[19,20,21,22]
**Inguinal hernia**	70–95%	[12,19]	60–75%	[19,20,21,22]
**Hepatosplenomegaly**	60–90%	[10,12,19,23]	70–85%	[19,20]
**Skeletal manifestations**	80%	[24]	80%	[22,25]
**Kyphosis**	34%	[10,12,18,19,23,24]	70–90%	[26]
**Odontoid hypoplasia**	rare	[10]	65%	[27]
**Joint stiffness**	75–90%	[10,19,23,24]	93%	[19,20,26]
**Poor growth**	79%	[12,19,24]	100%	[19,22,24]
**Epidermal symptoms** **(thickened skin with pebble formation, persistent Mongolian spots)**	13–17%	[19,28,29,30]	rare	
**Coarse facial features**	95%	[10,23]	86–97%	[13]
**Upper respiratory issues**	100%	[12,19]	80–100%	[19,20,22,25]
**Lower respiratory issues**	80–90%	[12,24]	80–90%	[21,22,31]
**Loss of hearing**	70–95%	[8,12,28,32]	76–100%	[20,25]
**Valvular heart disease**	50–60%	[10,23,28,33,34]	40–100%	[13,20,21,22,25,35,36]
**Corneal clouding**	rare	[19]	71–88%	[19,20,21,22,25]
**Seizures**	60%	[12,37]	rare	[38]
**Cognitive impairment**	100%	[12,23,28]	100%	[22,39,40]
**Behavioral disturbances**	30–45%	[8,12,19,23,28,41]	rare	[19]
**Diarrhea**	60%	[12,19,23,28]	rare	[19]

**Table 2:** Earliest time for manifestations in patients with MPS IH and MPS IIA.

	MPS IH	MPS IIA
**Cognitive development**	Progressive cognitive decline beginning at age 6–15 months [18,39,176,177,178]	Normal until age 3–4 years, followed by plateau and rapid decline [4,179]
**Coarse facial features**	6–12 months [18]	2–4 years [23]
**Hearing loss**	6–12 months [20]	4 years [32]
**Cardiac valve disease**	1 year [18]	6 years [23]
**Kyphosis**	1 year [18]	6 years [23,24]
**Axial growth**	Normal until age 3 years	Normal to accelerated until age 5–6 years [23,180]
**Average age at diagnosis**	1 year [18]	3–4 years [23,24,180,181]

**Table 3:** Earliest time for manifestations in MPS I and MPS II mice.

	MPS I	MPS II
Behavior (hypoactivity)	2–4 months [182,183]	6–10 months [160,164,166,168]
Zygomatic arch width	Increased at 4 weeks [184]	Increased at 2–3 months [164,185]
Hearing loss	2 months [186]	17 weeks [163]
Elevated GAG levels in urine and tissue	4 weeks [173,184,187]	4–6 weeks [162,164,165,167,185]

**Table 4:** Outcome of HSCT and ERT on manifestations in patients with MPS II.

Manifestation	ERT	HSCT
**Hepatosplenomegaly**	Improved [192,199,200,201,202,203,204,205]	Improved [206,207] Improved (case studies *n* < 10) [208,209,210]
**Skeletal manifestations**	No change [204]	No change (case study *n* = 1) [208] Improved (case study *n* = 1) [198]
**Poor growth**	Minimal effect [199,211]	Improved [212] Improved (case study *n* = 1) [198]
**Coarse facial features**	Improved [201]	Improved [206] Improved (case studies *n* < 10) [208,210,213,214]
**Upper respiratory function**	Improved [205]	Improved [206,207]
**Lower respiratory function**	Improved [191,199,201,203]	NA
**Heart hypertrophy**	Improved [191,194,199]	Improved [215]
**Valvular heart disease**	Prevention (when administered very early) [199] No change [204]	Stabilization/improved [206,215] Improved (case studies *n* < 10) [209,216,217]
**Joint stiffness**	No change [199,200] Improved (shoulders) [195,205]	No change [206] No change (case studies *n* < 10) [214,216,218,219] Improved [207,220] Improved (case studies *n* < 10) [208,209,213]
**Endurance**	Improved [192,194,195,201]	Improved [217]
**Skin, thickened with pebble**	Improved [199,221,222]	Improved (case studies *n* < 10)[214,216,218,223]
**Cognitive impairment**	No change [195,201]	Improved [217] Improved (case studies <10) [198,217] Worsening (case studies *n* < 10) [208,209,210,219,224]
**Apnea**	Improved/stabilized apnea (obstructive) [182]	Improved apnea (not clarified whether obstructive or central) [207]
**Diarrhea**	Improved/stabilized [205]	NA
**Activity of daily living (ADL)**	NA	Improved [207,215,225]
